# Age Standardization in Health Statistics: History and Future Perspectives

**DOI:** 10.2188/jea.JE20250668

**Published:** 2026-11-05

**Authors:** Kota Katanoda, Freddie Bray, Eric J. Feuer, Angela B. Mariotto, Hirokazu Tanaka, Aesun Shin, Kyu-Won Jung, Tomohiro Matsuda

**Affiliations:** 1 National Cancer Center Institute for Cancer Control, Tokyo, Japan; 2 Cancer Surveillance Branch, The International Agency for Research on Cancer (IARC), Lyon, France; 3 Division of Cancer Control and Population Sciences, National Cancer Institute, Bethesda, MD, USA (EJF-contractor); 4 Department of Preventive Medicine, Seoul National University College of Medicine, Seoul, Korea; 5 Korea Central Cancer Registry, National Cancer Center, Goyang, Korea

**Keywords:** health statistics, age-standardization, risk adjustment, demographic aging, population statistics

## Abstract

Age standardization is a key statistical method used in health statistics to adjust rates, such as mortality or incidence, enabling comparisons across populations or time points with different age structures. This review traces its historical development, global and country-specific practices, and future directions. The method dates back to the 19th century, with major adoption in the 20th century through Segi and Doll’s World Standard Population. While the World Health Organization (WHO) introduced an updated standard in 2000, the International Agency for Research on Cancer (IARC) continues to use Segi and Doll’s standard in the Cancer Incidence in Five Continents series, prioritizing consistency and comparability in long-term cancer surveillance. Case studies from the IARC, the United States, Japan, and the Republic of Korea (Korea) illustrate different responses to changing demographics. The United States adopted the 2000 standard with expanded age detail for the elderly population. Japan introduced the 2015 Japan Standard Population to account for its rapidly aging society, though regional data limitations presented challenges. Korea, experiencing one of the fastest aging transitions globally, updated to a 2020 standard for more accurate national and sub-national reporting. The review also emphasizes that age standardization can obscure important age-specific trends. Methods like Joinpoint clustering help detect divergent trends by age groups. Looking forward, age standardization remains essential amid global demographic shifts. However, updates of standard populations must balance improved relevance with the need for continuity and robust data. International coordination and digital tools will support more flexible and transparent health statistics in the future.

## INTRODUCTION

Age standardization is a basic procedure for health statistics, which is defined as “a procedure for adjusting rates (eg, death rates) designed to minimize the effects of differences in age composition in comparing rates for different populations”.^1^ The original concept involved deriving a crude rate that would be observed in a given cohort if its age distribution was the same as that of a “standard population”.^2^ Although a similar measure called “comparative mortality figure”, which is expressed as a ratio between the rates of target and reference populations, was introduced,^2^ age-standardized rates have predominantly been used in health statistics. Perhaps the most widely applied “standard population” is the World Population of Segi and Doll, which was proposed by Japanese researcher Dr Mitsuo Segi (1908–1982) in 1960 to compare cancer statistics based on an aggregation of population data from 46 countries,^3^ and simplified by rounding off the age-specific weights in the first Volume of Cancer Incidence in Five Continents (CI5) by Sir Richard Doll et al.^4^ Age-standardized rates or numbers have also been used to describe population-based disease burdens other than cancer. This method of age standardization is known as the “direct” method; we focus on this method in this review rather than the “indirect” approach, and for simplicity, we use the term “age standardization” as a synonym of the direct method from hereon in.

While individual investigators or organizations have sought to periodically establish “new” standard populations updated on the basis of demographic change (population ageing and growth), such ad hoc standard populations, may lead to considerable effort replacing prior published rates standardized to a previous standard. The age-standardized rate is, after all, intended as a summary rate based on the pattern of age-specific rates in a population with the age composition of the standard. The utility of age standardization lies in the ability to compare populations (or a single population over time) and estimate of the relative risk of disease (here, “relative risk” refers to the background difference in mortality/incidence rates) between the compared populations as a ratio of age-standardized rates. Age standardization has many applications but also limitations in characterizing summary rates between populations and or in tracking trends over time. In this review, we aimed to provide an international history of age standardization for health statistics and its future perspectives.

## BRIEF HISTORY

Historically, Neison is said to be the first to introduce the concept of age standardization in 1844.^5^^,^^6^ As official statistics, the Registrar General’s report of 1883 in England and Wales was the first to use of an age-standardization method.^5^ Internationally, it was used by Percy Stocks in 1959 to compare summary cancer incidence rates across different countries based on six age categories.^7^ As stated above, Segi and Doll et al proposed a standard population which had long been called “World Health Organization (WHO) World Standard Population”. The main feature of this standard population is the usage of 18 age categories and greater weights attributed to younger age groups reflecting typical age distributions of many regions, especially in low- or middle-income countries, at that time (Figure 1). The WHO had been officially using this standard population until it was replaced with a new one (WHO 2000–2025 Standard Population) in 2001.^5^ In this revision, a different age categorization was adopted, separating the age 0 year from 1–4 years and breaking down the age groups of 85 years and older. It also reflected a global change in age distribution, which slightly shifted toward older age groups. The change has however been questioned by Bray et al, given that the ratios of rates age-standardized using the standard World Population of Segi and Doll are as good approximations of the relative risks of different cancer types as the WHO 2000–2025 Standard Population,^8^ and the International Agency for Research on Cancer (IARC) has continued to use the former for summary comparisons of cancer incidence rates (eg, in successive volumes of the high-quality cancer registry compilation, CI5).^9^

**Figure 1.**
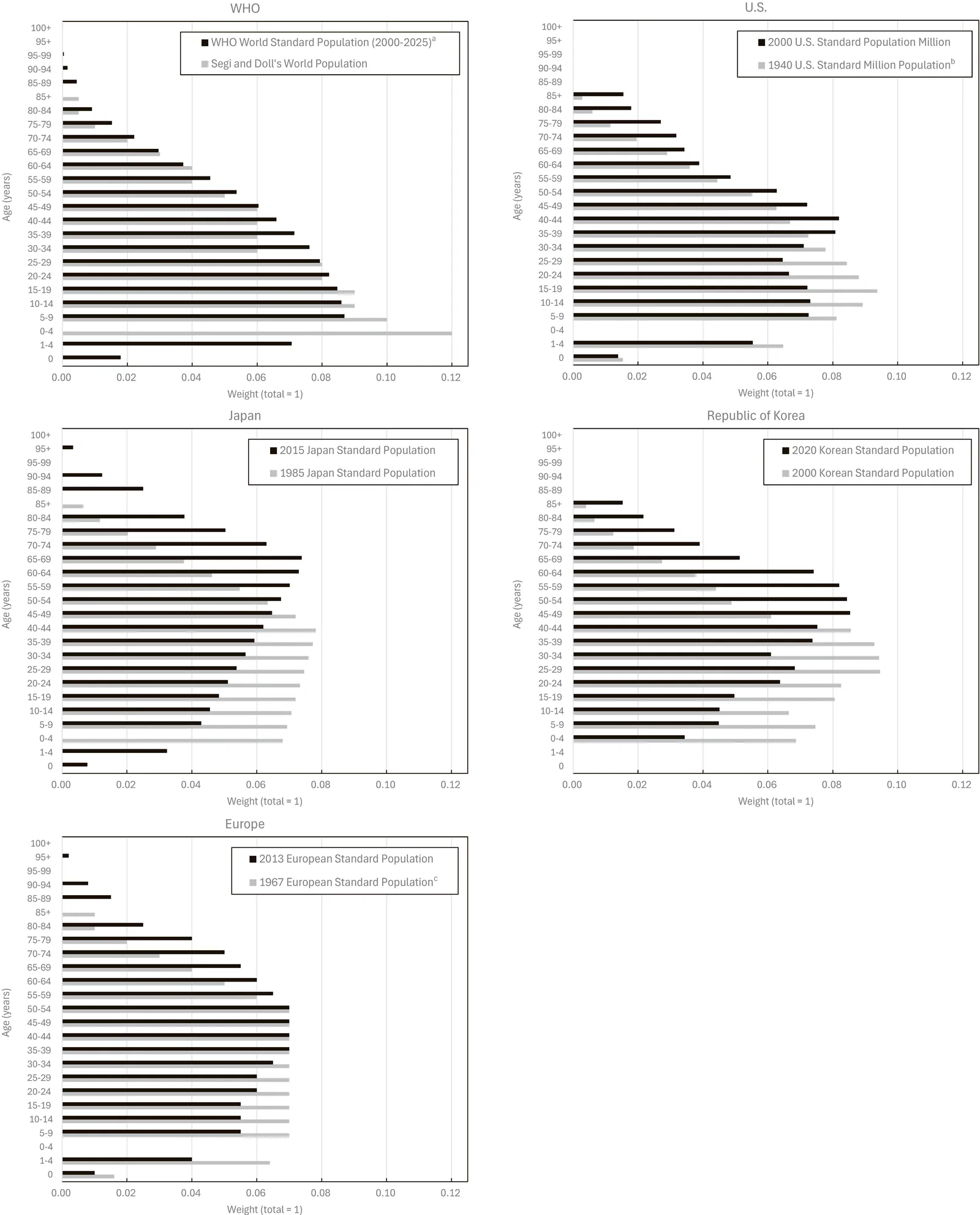
Standard populations of each country/organization, expressed as weights (total = 1) a. Modified version by the Surveillance, Epidemiology, and End Results (SEER), National Cancer Institute, U.S. (https://seer.cancer.gov/stdpopulations/stdpop.singleages.html) b. Caution: The 1940 US Standard Million Population officially uses 10-year groupings for ages (5–14, 15–24, … , 75–84 years). Five-year weights are displayed only for consistency in comparing to other standard populations, but the 1940 US Standard Million Population does not utilize these age groupings. c. This European Standard Population was originally prposed in 1967 (Ref. 10), but is also notated as “1976 European Standard Population (Ref. 11)” WHO: World Health Organization, U.S.: United States

There have also been region-specific standard populations. For example, Doll and Cook proposed African and European standard populations in 1967 based on 18 age categories.^10^ The European Standard Population was updated in 2013, which also introduced a detailed age categorization for the age groups of 85 years and older and reflected more recent (ie, aging) age distribution.^11^ The United States has also developed its own standard population and updated it to the current United States 2000 Standard, beginning with data reported for 1999. Regarding the latest changes in Europe and the United States, many discussions exist about the effects on statistics in the context of specific diseases, such as cancer and cardiovascular diseases, as well as health disparities.^12^^–^^16^ Many other countries developed their own standard populations, as described below.

## CONCEPT AND PURPOSE OF AGE-STANDARDIZATION

An age-standardized rate (ASR) is defined as a weighted average of age-specific crude rates. Mathematically the ASR (per 100,000 population) for an age group comprised from ages *x* through *y* is calculated as below, where *n_i_* denotes the number of events (eg, incidence or deaths) for the age group *i*, *p_i_* denotes the population, and *w**_i_* denotes the weight for the same age group.

$${ASR}_{x-y}=\sum_{i=x}^{y}\left[\left(\frac{n_{i}}{p_{i}}\right)\times 100,000\times w_{i}\right]$$

The weight *w**_i_* is calculated using the following equation, where *stdpop_i_* (or *stdpop_j_*) denotes the age-specific population of the age group *i* (or *j*).

$$w_{i}=\frac{{stdpop}_{i}}{\sum_{j=x}^{y}{stdpop}_{j}}$$

The standard error of the ASR can also be calculated based on the assumption of the distributions to give the confidence interval.^17^^,^^18^

Conceptually, the ASR is the expected number of events if the age-specific rates of a given population occurred in a population which had the same age distribution as the standard population. That is why it is used to compare disease rates in different regions or time points minimizing the effect of differences or changes in age composition. As a weighted mean, it summarizes the patterns of age-specific rates and so inevitably reduces the information about differential features of each age category.

For the calculation of ASRs, age-specific data are required both for the number of events (numerators) and populations (denominators) for all age groups. If the standard population uses detailed age categories for 85 years or older (eg, 85–89, 90–94, and ≥95 years), detailed data for those age categories are required not only for the number of events but also for the populations. Therefore, when we change the standard population, it is essential to prepare those data for the target populations and time periods.

## CASE HISTORIES

### IARC

Documenting variations in cancer incidence across countries and over time is a critical method for demonstrating the avoidability of human cancer.^19^ To enable valid comparisons between populations with differing age structures, incidence rates must be age-standardized. The conceptual foundation for such summary measures was first established at a 1965 sub-committee meeting of the International Union Against Cancer, the same year the IARC was founded. This meeting proposed three regional standard populations—African, European, and a “world standard” derived by Segi and Doll (Figure 1).^3^^,^^10^ All three were subsequently employed in the inaugural volume of CI5.^4^

IARC subsequently adopted Segi and Doll’s world standard as the reference for the CI5 series, including the present Volume XII.^20^ This allowed for the direct comparison of incidence rates from diverse registries (eg, Colombia, Denmark, and Japan) both within and between volumes. While Segi and Doll’s standard became a cornerstone for international cancer risk assessment, its underlying population weights were increasingly seen by some as outdated, failing to represent contemporary and future global populations characterized by longer life expectancy and ageing. In response, the WHO introduced a new “World Standard Population” around the year 2000, based on the United Nations-projected average global age structure for 2000–2025 (Figure 1), which features a substantially larger proportion of older individuals.^5^

#### Are standard populations in need of updating?

The primary objective of age standardization is to remove the confounding effect of age to facilitate comparison, not to approximate the crude rate. From IARC’s perspective, the critical question was how well the ratio of ASRs approximates the age-adjusted relative risk. An IARC analysis using 1970–1994 incidence data for four common cancers across demographically contrasting populations found no statistically significant difference in relative risk estimates between Segi and Doll’s and WHO standards.^8^ Consequently, it was concluded in 2002 that Segi and Doll’s standard performed as well as the WHO standard, and that the drawbacks of transitioning—including potential for misinterpretation, delayed reporting, and resource demands—outweighed the benefits.

However, this analysis did not address other important considerations, such as whether the choice of a global standard affects the ranking of ASRs for different cancers, which could influence cancer control prioritization. Furthermore, the practical challenges of adopting a new standard have likely been mitigated by decades of advances in web-based computational technology. A web-based data preparation tool for age-specific populations and numbers of health outcome events would make it easier to adopt a new standard population. Therefore, it would be valuable to revisit this question with more recent data to determine if the earlier conclusions remain robust.

### United States

Since 1943, the National Center of Health Statistics (NCHS), the United States federal agency which is part of the Centers for Disease Control (CDC) and coordinates health statistics for the United States government) and the individual states (who collect death certificates for state reporting and reports them to the NCHS for national reporting) had used a 1940 United States standard million population for age-adjustment (Figure 1, eTable 1).^21^ When reporting rates for 1940 using this standard, the age-adjusted rate equals the crude rate, and it is generally desirable that the range of years reported not have population weights that stray too dramatically from the standard. By the 1990’s it was felt that the 1940 standard was becoming “out of date” in the sense that the United States population was now more heavily weighted towards an older population, making the age-adjusted rates quite different than the crude rates. This was reflected by the fact that at least three other standards were being used by United States federal and state agencies. For example, the National Center for Chronic Disease Prevention and Health Promotion, CDC reported United States trends in ischemic heart disease deaths from 1990–1994 using a 1980 United States standard,^22^ and the National Cancer Institute (NCI) itself used at least three different standards: cancer incidence and mortality rates for annual reports used a 1970 United States standard^23^; the cancer institute’s goals for 2000, developed in 1985,^24^ were reported using the 1980 United States standard; and for the United States atlas of cancer mortality 1950–1980, a 1960 United States standard was used.^25^^,^^26^ It was recognized that the use of a single more contemporary standard by federal agencies would help alleviate misunderstandings, and would make the reported age-standardized rates generally more consistent with crude rates. As a result of two workshops sponsored by NCHS in 1991 and 1997,^27^^,^^28^ federal agencies adopted the current United States 2000 standard beginning with data reported for 1999 and after (Figure 1, eTable 1). There are no current plans underway to change the standard.

#### Consistency problems in the 2000 standard caused by rounding when computing a standard million

The new 2000 standard million was originally published as 11 age groups (0, 1–4, 5–14, 15–24, 25–34,… 75–84, ≥85 years) and was subsequently expanded to 19 age groups (0, 1–4, 5–9,…. 80–84, ≥85 years) for use in cancer surveillance. To allow for flexibility of standardization in many different age groupings (eg, adult tobacco use is often reported for those aged 18 years and above), or even single year of age standardization, the NCI obtained the same 2000 standard population used by NCHS based on unpublished data used by United States Census Bureau for their population projections.^29^ This projected population of 274,633,642 now provided in single years of age, was then rounded to form a standard million in single years of age, but when summed did not match the NCHS standard in 11 age groups. It was decided that to allow for consistency and precision across different age groupings, the traditional standard million population would be replaced by a standard population age distribution in single years of age representing the actual projected total population of the United States (reference and eTable 2).^30^ The result is a standard age distribution that can be aggregated as needed while maintaining consistency across applications. This revised 2000 standard was accepted by the NCHS beginning with 2003 data for use by federal health agencies.

#### Increasing the number of age groups used for age adjustment to reflect the changing age distribution among the oldest Americans

The number of adults aged 85 years and older has increased from 4.3 million in 2000 to 6.3 million in 2020 and is projected to nearly quadruple between 2000 and 2040 in the United States.^31^ This substantial growth in the ≥85 years population has significant implications for healthcare, social services, and the economy as a whole. Of those people aged ≥85 years in 2000, 34% were ≥90 years, while 40% were ≥90 years in 2020. Because of increasing interest at the NCI of cancer in elderly populations, starting with the April 2025 release of cancer surveillance data, the default age groups for age adjustment include more detailed breakdown for the ≥85 years population: 85–89 and ≥90 years, when using 5-year age group data, or single-year ages (85, 86, 87, 88, 89, and ≥90 years) when individual age data is available (eTable 2). The standard populations used for age adjustment remain based on the same revised 2000 United States Census estimates described above.^30^^,^^32^ Subdividing the ≥85 years population into finer age groups by the NCI acknowledges the diversity within this demographic. These updates improve the ability of age-standardized estimates to adjust for changes in the age distribution of those aged 85 years and older and enhances the ability to identify and analyze trends among the oldest. The population data for the calculation of age-standardized rates are provided in a consistent form by the United States Census Bureau. If various agencies throughout the government have needs that are not being met by the United States Census Bureau, after discussions between the various agencies and the United States Census Bureau, the work can potentially be procured through an interagency agreement.

### Japan

In Japan, the National Vital Statistics was launched in 1899 and continued for over 120 years, including the chaotic period of the World War II around the 1940s. The most common mortality statistics are those from 1950 onwards due to the historical background of public health in Japan (eg, Public Health Center Act established in 1947) and the stability of the data. They are used for demographics, health reports, administrative documents, professional trainings, and other purposes. The Ministry of Health, Labour and Welfare (MHLW) developed and used the Japanese standard populations for age standardization of mortality. The Japanese standard population has changed from “1935 Japan Standard Population” to “1985 Japan Standard Population” and the current version of “2015 Japan Standard Population” over a half-century.^33^^,^^34^

The first version, “1935 Japan Standard Population,” was used from 1951 through 1989 for the official reports of the National Vital Statistics. The “1935 Japan Standard Population” was defined as the age distribution of the 1935 Population Census and used only by MHLW to calculate age-standardized mortality; therefore, the details of the standard population had not been published for researchers and public health practitioners. At that time, age-standardized mortality was called “corrected mortality” in Japanese; however, the terminology has been unified into “age-standardized mortality” (or “age-adjusted mortality”) since 1990 because this Japanese terminology confuses the interpretations. In 1990, the “1985 Japan Standard Population” was introduced because the “1935 Japan Standard Population” significantly differs from the actual population distribution due to changes in the population structure (ie, population aging).^33^ The “1985 Japan Standard Population”, including the oldest aggregated age category of “85 years and over”, was developed based on the 1985 Population Census using the 1,000-person unit, with corrections for extreme fluctuations due to the baby boom. The “1985 Japan Standard Population” has been widely used for three decades, and the established age-standardized mortality rates are the most common and familiar health statistics for public health practitioners and medical professionals. In 2022, the MHLW introduced “the 2015 Japan Standard Population”.^35^ Simultaneously, the MHLW officially abolished the publication of official health statistics using the “1985 Japan Standard Population” in 2022. For 2020 and later, national mortality statistics have officially been provided using only the new standard population. However, almost all age-standardization (for mortality or incidence) can be calculated by applying the “1985 Japan Standard Population”, and this is still used to set policy targets in many settings in the 2020s. For example, the National Cancer Center, Japan, provides cancer mortality statistics using both “1985 Japan Standard Population” and “the 2015 Japan Standard Population” since 2022.^34^ The motivation for updating the standard population was to deal with the drastic changes in the age structure of the Japanese population since 1985. A feature of the 2015 Japan Standard Population is that it includes greater weights on older population categories than those of other standard populations, such as the 2013 European Standard Population, 2000 United States Standard Population, and WHO World Standard Population (2000–2025).^36^ To the best of our knowledge, this is the “oldest” standard population globally (Figure 1). Another important feature is the change in the oldest aggregated age category from “85 years and over” in the “1985 Japan Standard Population” to “95 years and over” in the “2015 Japan Standard Population”. This enables the analysis, including detailed older age groups (eg, “85–89 years”, “90–94 years”) into age standardization.

We carefully assessed the practical implications of the update to the 2015 Japan Standard Population because the latest updates may cause practical problems, such as the sustainability of evaluations across different time points and regional statistics. We concluded that the effect of introducing the 2015 Japan Standard Population for interpreting trends in all-cause mortality was considered minimal.^36^ This is because the trends in crude mortality rates by each 5-year age category were consistent (continued declines) over a half-century for the Japanese population. However, we found that the ability to conduct a complete analysis was dependent on the availability of population data, especially for detailed older age groups (eg, “85–89 years”, “90–94 years”). In general, the number of deaths is available for older age groups (eg, 90–94 years) in different time points and prefecture level because the National Vital Statistics includes the oldest aggregated age category of “100 years and over” since 1950. However, prefecture-level population data is not surely available for the exact age categories used in the “2015 Japan Standard Population”, especially for older age groups (eg, 90–94 years).^37^ This hinders smooth switching to the new standard population at prefecture-level mortality. Alternatively, it is possible that older populations were aggregated into a single “85 years and older” group using the “2015 Japan Standard Population”. However, this approach violates the original concept of a standard population and confuses results with a patchwork of different versions of the standard population.

A new standard population in response to rapid changes in the age structure is required; however, updates could be confusing if insufficient population and mortality data cover these updates. Japan experienced a lack of population data for older age groups at the prefecture level. Japan’s lessons underscore that improving population data is key for future standard population updates worldwide.

### Republic of Korea (Korea)

Korea has undergone one of the most rapid demographic transitions in the world. Korea entered a super-aged society in 2024, with over 20% of its population aged 65 years and older, and it rapidly transitioned from an aging society with over 7% of the population aged 65 years and older (2000) to an aged society with 14% of the population aged 65 years and older (2017) to a super-aged society (2024), while fertility rates fell to the lowest levels globally.^38^ This dramatic change in age structure has highlighted the essential role of ASRs in disease surveillance and policy planning. However, Korea’s experience also shows that the choice of a standard population can significantly alter the interpretation of health statistics over time.

In the 1990s, most health statistics in Korea relied on Segi and Doll’s world standard population, which places greater weight on younger age groups (Figure 1). While this supported international comparisons, it became less suitable for internal analyses as Korea’s population aged. In 1999, with the launch of national cancer statistics, the Korea Central Cancer Registry (KCCR) introduced the “Korean 2000 mid-year population” as the standard for domestic reporting (Figure 1). At the time, the age structure was still relatively young—age groups under 40 years accounted for about two-thirds of the population—making the 2000 standard a reasonable reflection of demographic reality.

By 2020, however, Korea’s population profile had changed significantly. The median age had risen from 31.8 years in 2000 to 43.7 years in 2020, and the share of adults aged 65 years and older had increased from 7.0% to 16.4%.^39^ Continuing to use the 2000 standard increasingly led to misleading assessments of disease burden. For example, in county-level analyses of breast cancer incidence, a region ranked first using the 2000 standard dropped to 12th when rates were recalculated with the 2020 standard, while another region rose from third to first.^40^ A similar effect was observed in suicide statistics: Korea’s 2010 ASR for suicide was the highest among Organization for Economic Co-operation and Development (OECD) countries using the OECD standard (35 per 100,000) but fell to 12th globally under the WHO standard (27.9 per 100,000), which places greater weight on younger age groups where Korea’s rates were relatively lower.^40^

To address these issues, the KCCR officially adopted the “Korean 2020 mid-year population” as the new national standard for sub-national cancer statistics, beginning with the 2022 release (Figure 1). Compared to the 2000 standard, the 2020 version reflects the ageing population by increasing the weights for older age groups and reducing those for younger ages. This update aimed to improve the relevance of statistics for current public health decision-making, even though it reduced comparability with historical data.

Implementing the new standard required substantial technical and communication efforts. KCCR reprocessed more than two decades of age-specific cancer data to ensure consistent time trends. In parallel, materials such as statistical dashboards, user guides, and metadata documentation were updated to help users interpret the new figures correctly. Mid-year population data are available for all sub-national districts and updated every year by Statistics Korea. Meanwhile, for global benchmarking, Korea continues to publish ASRs based on Segi and Doll’s standard, maintaining compatibility with international datasets.^3^^,^^10^

Korea’s experience demonstrates the need to align statistical assumptions with demographic realities, especially in fast-ageing societies. Regular review and timely updating of standard populations help preserve the accuracy, transparency, and policy value of health statistics.

## DETECTING DIFFERENT TRENDS ACROSS AGE GROUPS

Examining trends in age-adjusted rates for all ages combined may mask important differences in trends for specific age groups. In addition, the more there is a lack of parallelism (ie, inconsistency across age groups) between age specific trends, the more the age-adjusted trends for all ages combined will vary as a function of the selected age-adjustment standard. Conversely, if the age-specific trends are completely parallel, the trends will be completely invariant to the choice of the standard. Using cancer data as an example, one way of selecting age groupings for age-specific analyses is to use knowledge of how specific cancers progress or differ by age (ie, separating trends for female gynecologic cancers and female breast cancer into pre- and post-menopausal groups). In the absence of a strong biologic, epidemiologic, or demographic rationale to form specific age groupings, an alternative method is to use a data-driven algorithm to form clusters of adjacent age groups, where the age-specific trends within each cluster are as close to parallel as possible, and separating groupings where the trend differs. This has the advantage of letting the data speak for itself without biasing the results with preconceived notions about where the age breaks should occur. This serves the dual purpose of minimizing the chance of masking any age-specific trends when age adjusting, while having the resultant trends within a cluster be approximately invariant to the choice of the standard. Several tools have been provided by international and national agencies for the trend analysis of population-based incidence/mortality rates.^41^^,^^42^ The Joinpoint methodology is widely used to assist in the interpretation of cancer and other disease trends,^43^^,^^44^ which was extended to include a clustering algorithm to group contiguous age groups that are as close to parallel as possible within the grouping, and conversely not parallel across age groupings.^45^

This methodology is illustrated using United States male colorectal cancer mortality for 2000 through 2023 for men aged 30 and above. Figure 2A shows age-adjusted rates for the entire age range (age-adjusted using the United States 2000 standard population using 12 5-year age groups, and a single open-ended age group [ie, ≥90 years]) which shows a slowing in the rate of decline. In the United States, there has been a rise in colorectal cancer incidence among younger adults, and a decline among older adults. This rise among younger adults partly motivated the United States Preventive Services Task Force to change its recommendations to start screening at age 45 years instead of 50 years.^46^ Changes in trends in colorectal cancer mortality are of interest to understand the downstream impact of the changing incidence trends. The overall age-adjusted mortality rates mask this effect, so the Joinpoint clustering algorithm was used to show age groupings optimized so that the age-specific groups within each cluster are as close to parallel as possible. In this example, up to 10 clusters were allowed, and the algorithm examines all possible clusters of adjacent age groups (with clusters varying in size between 1 and 13 age-specific groups). Sometimes (especially when working with common disease and/or large populations) even small differences in parallelism yield different clusters. These differences may not be substantively important. For this reason, an optional criterion was added to the software to fit a model with fewer clusters if all pairs of adjacent age-grouping clusters do not meet a minimum Annual Percent Change (APC) difference in Joinpoint estimated slopes (expressed as an APC) averaged across all of the years of series. This optional criterion starts with the optimized number of clusters and steps down the number of clusters by one until the minimum APC difference is met for every set of adjacent clusters. In this example, using a minimum average APC difference of one percentage point resulted in three clusters (ages 30–54, 55–64, and 65–≥90 years) shown in Figure 2B, Figure 2C, and Figure 2D, respectively. The trends show a steady rise in the mortality rate since 2005 for those aged 30–54 years, a change in the trend from falling to rising in 2019 among those aged 55–64 years, and a steady decline in mortality for those aged 65 years and over. This software extension of Joinpoint was released in March 2026 (see https://surveillance.cancer.gov/help/joinpoint/statistical-notes/clustering-of-contiguous-age-groups/ for more information and updates on the release).

**Figure 2.**
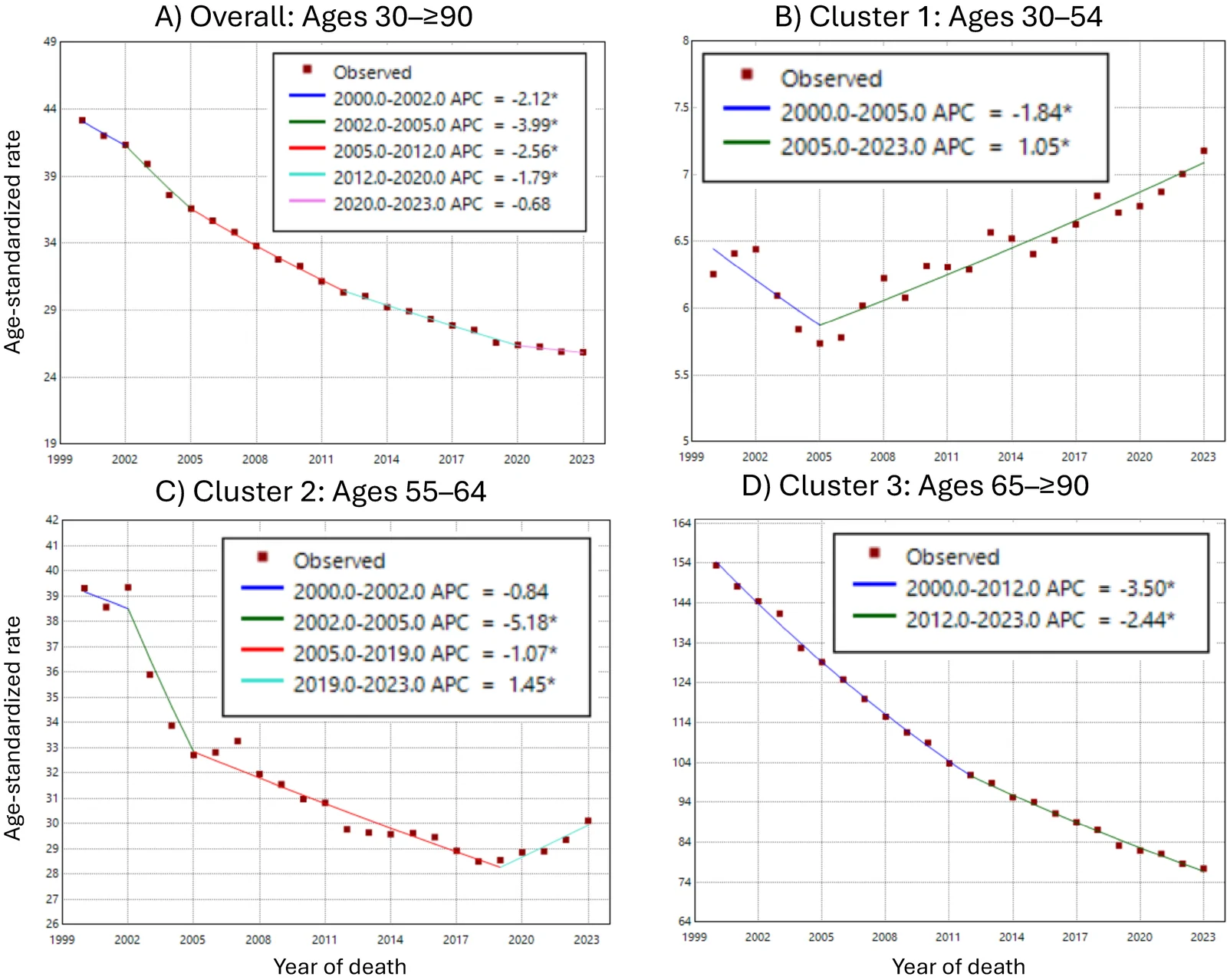
An example of different annual trends across age groups: U.S. male colorectal cancer mortality^a^ ^a^Age groupings selected using the Joinpoint clustering algorithm with the minimum difference worth detecting = 1%. ^*^Annual Percent Change (APC) significant (*P* < 0.05)

## FUTURE PERSPECTIVES

Figure 3 shows changes in population pyramids over 90 years from 1965, according to the World Bank income level.^38^ The differences in age structure have been becoming sharper, primarily due to the aging populations of high-income countries. These discrepancies are projected to persist in 2055, indicating that the age-standardizing methods currently in use will remain useful for decades to come. Age adjustment is necessary for examining past trends and predicting future disease burdens and formulating health policies. From this perspective, aligning the age structure with the present or future is crucial, and it is meaningful to consider updates every several decades, assuming the necessary data preparation is in place. The ASR is a hypothetical rate based on the assumption that the age structure is identical to that of a reference population. Therefore, it may have little practical significance to assume the age structure of a high-income country with a large elderly population onto a low-income country with a high birth rate and a large youth population, or vice versa. Accordingly, it would be reasonable to use a reference population whose age structure closely matches that of the target population.

**Figure 3.**
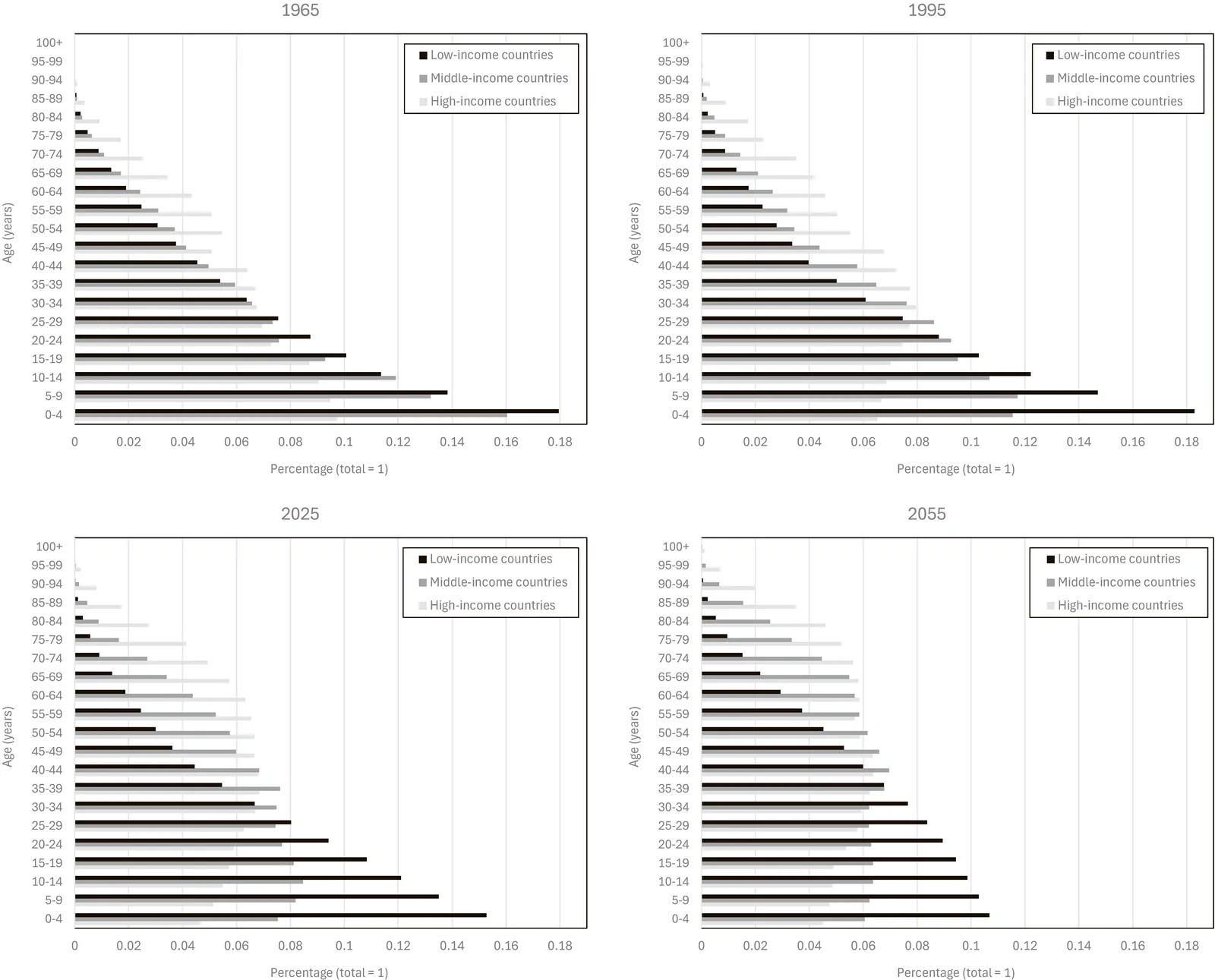
Estimated and prospected changes in population pyramids according to the World Bank income categories Source: United Nations, Department of Economic and Social Affairs, Population Division. World Population Prospects 2024, Online Edition. Population by Five-year Age Groups - Both Sexes (Medium variant). https://population.un.org/wpp/downloads?folder=Standard%20Projections&group=Population

Until now, the standard populations used for ASRs have been developed independently by each country or organization and updated as necessary. The primary reason for updates is changes in the age structure of the target population. A change in standard populations requires data preparation and recalculation. The change may result in a mere parallel shift in absolute values. Consequently, the decision to change should be based on balancing those benefits and costs. Utilizing web-based technology can potentially alleviate the burden of data replacement and recalculation. Preparing standards in single year age categories (rather than 5-year age categories) would allow for more flexible changes of age categorization, especially for older age groups (eg, a change from rounding ≥85 to ≥95 years old). Successful changes in standard populations depend on close collaboration among different health and statistics agencies for relevant data preparation. It should be noted again that ASRs only represent summary statistics across all age groups, assuming a certain age distribution. Using ASRs can lead to erroneous judgments when trends differ by age groups. A thorough examination from both summarized and disaggregated perspectives is crucial for temporal or spatial comparisons. We hope this review will contribute to better utilization of age standardization and further advancement of health statistics.

## SUPPLEMENTARY MATERIAL

eTable 1. Standard population used for each country/organization

eTable 2. 2000 U.S. Standard Population by single age
