# Supplementary material for: Age Standardization in Health Statistics: History and Future Perspectives
Source: J Epidemiol. 2026 Nov 5;36(11):337–45. doi: 10.2188/jea.JE20250668 (PMC13662267; doi:10.2188/jea.JE20250668)
Supplement: Supplementary file 1 [file je-36-337-s001.pdf]

eTable 1. Standard population used for each country/organization

Age categories	Segi and Doll's World Population	WHO World Standard Population (2000-2025)[a]	1940 U.S. Standard Million Population	2000 U.S. Standard Population Million	1985 Japan Standard Population	2015 Japan Standard Population	2000 Korean Standard Population	2020 Korean Standard Population	1967 European Standard Population[b]	2013 European Standard Population
Total	100,000	1,000,000	1,000,000	1,000,000	120,287	125,319,000	47,534,125	51,349,259.0	100,000	100,000
0 year	-	17,917	15,343	13,818	-	978,000	-	-	1,600	1,000
1-4 years	-	70,652	64,718	55,317	-	4,048,000	-	-	6,400	4,000
0-4 years	12,000	-		-	8,180	-	3,262,382	1,760,637.5	-	-
5-9 years	10,000	86,870	170,355 (5-14 years)	72,533	8,338	5,369,000	3,546,106	2,305,388.5	7,000	5,500
10-14 years	9,000	85,970		73,032	8,497	5,711,000	3,156,497	2,314,984.0	7,000	5,500
15-19 years	9,000	84,670	181,677 (15-24 years)	72,169	8,655	6,053,000	3,826,940	2,551,012.5	7,000	5,500
20-24 years	8,000	82,171		66,478	8,814	6,396,000	3,923,161	3,272,265.0	7,000	6,000
25-29 years	8,000	79,272	162,066 (25-34 years)	64,529	8,972	6,738,000	4,491,340	3,505,179.5	7,000	6,000
30-34 years	6,000	76,073		71,044	9,130	7,081,000	4,479,771	3,130,579.0	7,000	6,500
35-39 years	6,000	71,475	139,237 (35-44 years)	80,762	9,289	7,423,000	4,411,157	3,786,437.5	7,000	7,000
40-44 years	6,000	65,877		81,851	9,400	7,766,000	4,067,761	3,863,754.5	7,000	7,000
45-49 years	6,000	60,379	117,811 (45-54 years)	72,118	8,651	8,108,000	2,897,028	4,380,758.0	7,000	7,000
50-54 years	5,000	53,681		62,716	7,616	8,451,000	2,318,703	4,331,294.0	7,000	7,000
55-59 years	4,000	45,484	80,294 (55-64 years)	48,454	6,581	8,793,000	2,088,513	4,207,585.0	6,000	6,500
60-64 years	4,000	37,187		38,793	5,546	9,135,000	1,796,705	3,804,709.5	5,000	6,000
65-69 years	3,000	29,590	48,426 (65-74 years)	34,264	4,511	9,246,000	1,301,094	2,635,592.0	4,000	5,500
70-74 years	2,000	22,092		31,773	3,476	7,892,000	883,475	2,000,708.5	3,000	5,000
75-79 years	1,000	15,195	17,303 (75-84 years)	26,999	2,441	6,306,000	587,065	1,602,662.5	2,000	4,000
80-84 years	500	9,097		17,842	1,406	4,720,000	309,500	1,110,911.5	1,000	2,500
85+ years	500	-	2,770	15,508	784	-	186,927	784,800.0	1,000	-
85-89 years	-	4,398		-	-	3,134,000	-	-	-	1,500
90-94 years	-	1,500		-	-	1,548,000	-	-	-	800
95-99 years	-	400		-	-	-	-	-	-	-
≥95 years	-	-		-	-	423,000	-	-	-	200
≥100 years	-	50		-	-	-	-	-	-	-

[a] Modified version by the Surveillance, Epidemiology, and End Results (SEER), National Cancer Institute, U.S. (https://seer.cancer.gov/stdpopulations/stdpop.singleages.html)

[b] This European Standard Population was originally prposed in 1976 (Ref. X), but is also notated as "1976 European Standard Population (Ref. X)"

eTable 2. 2000 U.S. Standard Population by single age

	Single Ages to 89	Single Ages to 84
Total	274,633,642	274,633,642
00 years	3,794,901	3,794,901
01 years	3,758,562	3,758,562
02 years	3,773,025	3,773,025
03 years	3,791,001	3,791,001
04 years	3,869,031	3,869,031
05 years	3,896,081	3,896,081
06 years	3,917,855	3,917,855
07 years	3,978,143	3,978,143
08 years	3,903,983	3,903,983
09 years	4,223,778	4,223,778
10 years	4,230,322	4,230,322
11 years	4,027,959	4,027,959
12 years	3,941,299	3,941,299
13 years	3,923,270	3,923,270
14 years	3,933,929	3,933,929
15 years	3,952,423	3,952,423
16 years	3,853,629	3,853,629
17 years	4,012,263	4,012,263
18 years	3,936,904	3,936,904
19 years	4,064,299	4,064,299
20 years	4,037,599	4,037,599
21 years	3,764,802	3,764,802
22 years	3,555,718	3,555,718
23 years	3,489,233	3,489,233
24 years	3,409,873	3,409,873
25 years	3,421,099	3,421,099
26 years	3,328,203	3,328,203
27 years	3,434,987	3,434,987
28 years	3,450,602	3,450,602
29 years	4,087,176	4,087,176
30 years	3,999,004	3,999,004
31 years	3,810,183	3,810,183
32 years	3,774,385	3,774,385
33 years	3,840,938	3,840,938
34 years	4,086,860	4,086,860
35 years	4,288,078	4,288,078
36 years	4,349,620	4,349,620
37 years	4,469,476	4,469,476
38 years	4,290,207	4,290,207
39 years	4,782,575	4,782,575
40 years	4,666,685	4,666,685
41 years	4,493,582	4,493,582
42 years	4,487,560	4,487,560
43 years	4,424,004	4,424,004
44 years	4,407,398	4,407,398
45 years	4,268,017	4,268,017
46 years	4,033,859	4,033,859
47 years	3,958,468	3,958,468
48 years	3,681,489	3,681,489
49 years	3,863,960	3,863,960
50 years	3,720,935	3,720,935
51 years	3,504,329	3,504,329
52 years	3,475,657	3,475,657
53 years	3,754,218	3,754,218
54 years	2,769,220	2,769,220
55 years	2,749,739	2,749,739
56 years	2,786,795	2,786,795
57 years	2,947,472	2,947,472
58 years	2,404,462	2,404,462
59 years	2,418,766	2,418,766
60 years	2,259,141	2,259,141
61 years	2,179,759	2,179,759
62 years	2,132,873	2,132,873
63 years	2,030,730	2,030,730
64 years	2,051,769	2,051,769
65 years	2,033,933	2,033,933
66 years	1,862,107	1,862,107
67 years	1,849,893	1,849,893
68 years	1,788,769	1,788,769
69 years	1,875,238	1,875,238
70 years	1,843,087	1,843,087
71 years	1,784,744	1,784,744
72 years	1,802,080	1,802,080
73 years	1,674,285	1,674,285
74 years	1,621,378	1,621,378
75 years	1,610,943	1,610,943
76 years	1,530,137	1,530,137
77 years	1,450,062	1,450,062
78 years	1,456,186	1,456,186
79 years	1,367,231	1,367,231
80 years	1,172,978	1,172,978
81 years	1,065,672	1,065,672
82 years	963,587	963,587
83 years	890,893	890,893
84 years	807,104	807,104
85/≥85 years	693,158	4,259,173
86 years	607,940	
87 years	536,762	
88 years	452,814	
89 years	387,893	
≥90 years	1,580,606	

Source: Standard Populations - Single Ages, Surveillance, Epidemiology, and End Results (SEER), National Cancer Institute, U.S. (https://seer.cancer.gov/stdpopulations/stdpop.singleages.html)
